# Diagnostic Value of Semiquantitative Analysis of Dynamic Susceptibility Contrast Magnetic Resonance Imaging with GD-EOB-DTPA in Focal Liver Lesions Characterization: A Feasibility Study

**DOI:** 10.1155/2015/630273

**Published:** 2015-05-06

**Authors:** Davide Ippolito, Maddalena Colombo, Chiara Trattenero, Pietro Andrea Bonaffini, Cammillo Talei Franzesi, Davide Fior, Sandro Sironi

**Affiliations:** ^1^School of Medicine, University of Milano-Bicocca, 20900 Milan, Italy; ^2^Department of Diagnostic Radiology, H. S. Gerardo Monza, Milan, Italy

## Abstract

*Purpose.* To assess the diagnostic accuracy of dynamic susceptibility contrast-enhanced magnetic resonance imaging (DSCE-MRI) in differentiation between benign and malignant liver lesions by assessment of tumoral perfusion parameters. *Methods Materials.* Seventy-three patients with known focal liver lesions, including 45 benign (16 FNH, 27 angiomas, and 2 abscesses) and 28 malignant ones (17 metastases, 9 HCCs, and 2 cholangiocarcinoma) underwent 1.5 T MRI upper abdominal study, with standard protocol that included dynamic contrast-enhanced sequences. On dedicated workstation, time-intensity curves were determined and the following perfusion parameters were calculated: relative arterial, venous and late enhancement (RAE, RVE, RLE), maximum enhancement (ME), relative enhancement (RE), and time to peak (TTP). *Results.* All diagnoses were established either by histopathology or imaging follow-up. Perfusion mean values calculated in benign lesions were RAE 33.8%, RVE 66.03%, RLE 80.63%, ME 776.00%, MRE 86.27%, and TTP 146.95 sec. Corresponding perfusion values calculated in malignant lesions were RAE 22.47%, RVE 40.54%, RLE 47.52%, ME 448.78%, MRE 49.85%, and TTP 183.79 sec. Statistical difference (*p* < 0.05) was achieved in all the perfusion parameters calculated, obtaining different cluster of perfusion kinetics between benign and malignant lesions. *Conclusions.* DSCE-MRI depicts kinetic differences in perfusion parameters among the different common liver lesions, related to tumour supply and microvascular characteristics.

## 1. Introduction

A variety of pathologic conditions such as benign and malignant neoplasms, abscesses, and angiomas may occur in the liver. Their detection and characterization have profound implications for patients' prognosis and treatment strategies [1].

Hepatocellular carcinoma (HCC) is the most common primary liver cancer and is responsible for more than one-half million deaths annually worldwide [2]; therefore, its early detection may be critical to patient outcome. Liver is also the most common site of metastatic disease from different primary malignancies, including colorectal, lung, breast, and renal cancer. Discrimination between benign cavernous hemangiomas and HCC or metastases is often difficult. Focal nodular hyperplasia (FNH) is the second most common benign liver tumours after angiomas; even its differentiation with other hypervascular lesions (adenoma, HCC, and metastases) might be challenging because FNH and hepatic adenoma (HA) easily mimic malignant hepatic tumours [3]. Therefore, the ability to accurately diagnose liver lesions, to treat malignant ones, and to monitor their response at follow-up is essential for the proper management of patients, especially oncologic ones [4].

The potential benefits of higher sensitivity and specificity in morphological and functional liver imaging have substantially increased in recent years. Because most pathologic entities of the liver affect blood flow regionally, globally, or both [5], perfusion techniques have been invoked as a means of further improving the sensitivity and specificity of diagnostic liver imaging [1]. Angiogenesis represents the development of new blood vessels from the existing vascular bed and is considered essential for tumour growth, tissue invasion, and metastases. Hence, study of this process is expected to help in the design of new treatment strategies for several types of malignancies [6, 7]. Indirect and noninvasive imaging of angiogenesis in the liver is most commonly performed employing two main approaches that include multidetector computed tomography (MDCT) and magnetic resonance (MR) imaging. Recently, perfusion-weighted imaging (PWI) of the liver has shown the potential of furnishing additional tools to assess liver function, providing information concerning both the soft tissue characteristics and the vascularity (perfusion and angiogenetic activity) of the lesions [8]. With recent advances in MR imaging technique, including high performance gradients and parallel imaging, it is now possible to cover the entire liver volume, with good spatial and temporal resolutions. Moreover, MR imaging has several advantages over CT including the lack of ionizing radiation. Therefore, it has the ability to image whole organs repeatedly and dynamically and the possibility of repeating the perfusion study several times after treatment [9].

The purpose of this study was to assess the feasibility of dynamic susceptibility contrast-enhanced magnetic resonance imaging (DSCE-MRI) in the differentiation between benign and malignant liver lesions by the noninvasive assessment of quantitative tumoral kinetics.

## 2. Materials and Methods

### 2.1. Study Population

A series of 73 patients (30 women and 43 men; age range: 18–84 years; mean age 62.26 years) with known or suspected focal liver lesions, including 45 benign (16 FNH, 27 angiomas, and 2 abscesses) and 28 malignant ones (17 hypovascular metastases, 9 HCCs, and 2 cholangiocarcinomas) were retrospectively evaluated. Lesions' diagnosis either was pathologically confirmed or was rendered on the basis of radiologic findings from contrast-enhanced ultrasonography (CEUS), MDCT, and MRI (including hepatobiliary phase study). We excluded MR studies, which were incomplete or affected by severe motion artifact due to poor breath holding. Before underdoing MR examination, all subjects gave their informed consent to the administration of contrast medium, after the nature of the procedure had been fully explained.

### 2.2. MRI Protocol

All patients underwent an upper abdominal examination on a 1.5 T magnet (Achieva, Philips), using a 4-channel phased-array body coil, for both excitation and signal reception. At our institution routine MR liver imaging protocol includes axial T1-weighted in- and out-of-phase breath-hold spoiled gradient-echo (GRE), axial and coronal respiratory-triggered, fat-suppressed and turbo spin-echo (TSE), and T2-weighted and axial 3D T1-weighted fat-suppressed spoiled recalled-echo sequences (THRIVE). The dynamic study is obtained before and after the intravenous injection of 0.1 mL/kg of gadoxetic acid (Gd-EOB-DTPA, Primovist, Bayer Leverkusen, Germany), at a flow rate of 1.5 mL/sec, followed by a 30 mL saline flush at the same rate, using a power injector. Images are acquired in different phases and using bolus tracking (BT) technique to permit breath-hold coordination with contrast arrival at the level of celiac trunk, in order to acquire the arterial phase of hepatic enhancement. The portal venous and equilibrium phases were acquired after 60 and 140 sec, respectively, after the arterial phase. Sequences parameters used for DCE-MRI liver protocol are shown in Table 1.

### 2.3. Image Analysis and Quantification of Perfusion Parameters

Dynamic contrast-enhanced (DCE) raw data were transferred to an independent image workstation (Viewforum; Philips Medical Systems) with dedicated perfusion software (T1 Perfusion Package, Philips Medical Systems). Functional perfusion maps were generated and were displayed in a colour scale ranging from blue to red colour, blue colour being the lowest range of display for Relative Arterial Enhancement (RAE%), Relative Venous Enhancement (RVE%), Relative Late Enhancement (RLE%), Maximum Enhancement (ME%), Relative Enhancement (RE%), and Maximum Relative Enhancement (MRE%); red colour was the lowest range of display for time to peak (TTP, sec). RAE, RVE, and RLE represent the highest values percentage of intensity signal of contrast material concentration in the three different enhancement phases (arterial, venous, and delayed phase). ME and MRE represent the highest absolute values of intensity signal and TTP corresponds to the time to reach the maximum value of contrast material concentration.

For DCE, we applied the semiquantitative method that describes tissue enhancement using a number of descriptors derived from time-signal intensity curves (TSIC), as yet reported by Kambadakone and Sahani [10]. The time-signal intensity curves were automatically derived. RAE, RVE, RLE, ME, RE, MRE, and TTP were automatically calculated by manually drawing two different regions of interest (ROIs) both in the focal liver lesions (R1) and in the surrounding parenchyma (R2); both the ROIs were placed in a homogeneous region, avoiding vessels or artifacts. The resulting temporal changes in contrast enhancement were then analyzed to quantify a range of parameters that reflected the functional status of tissue perfusion.

### 2.4. Statistical Analysis

Mean and standard deviation values were used for descriptive purposes for all the perfusion parameters (RAE, RVE, RLE, ME, MRE, and TTP), calculated in the lesion and in the surrounding hepatic parenchyma, used as tissue reference. Commercial software (SPSS version 17.0; SPSS, Chicago, IL) was employed for statistical analysis. The Mann-Whitney test was used to evaluate if there were differences among each of the perfusion parameters between benign and malignant lesions, indicating *p* values < 0.05 as a statistically significant difference.

## 3. Results

**Figure 1:**
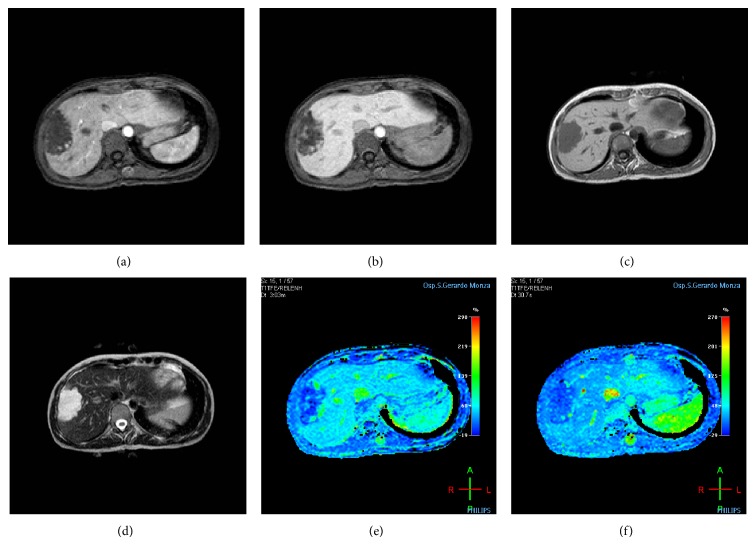
A 44-year-old man with gallbladder stones was evaluated. The MR imaging shows a focal lesion in the right lobe. (a, b) Axial arterial and delayed postcontrast injection images show irregular well-defined lesion, characterized by globular pattern enhancement at the periphery of the lesion in the arterial phase, with intensity signal that increases during delayed phase. (c) Axial T1-weighted in-phase echo-gradient sequence shows a homogeneous hypointense well-defined irregular nodular area in the seventh segment. (d) Axial T2 fat-sat weighted sequence shows well-defined nodular area, with high intensity signal. (e, f) On colour perfusion maps (Relative Arterial Enhancement and Relative Late Enhancement, resp.) the lesion corresponds to hypovascularized area, characterized by high vascularity hot-spots signals along the boundary of the lesion.

**Figure 2:**
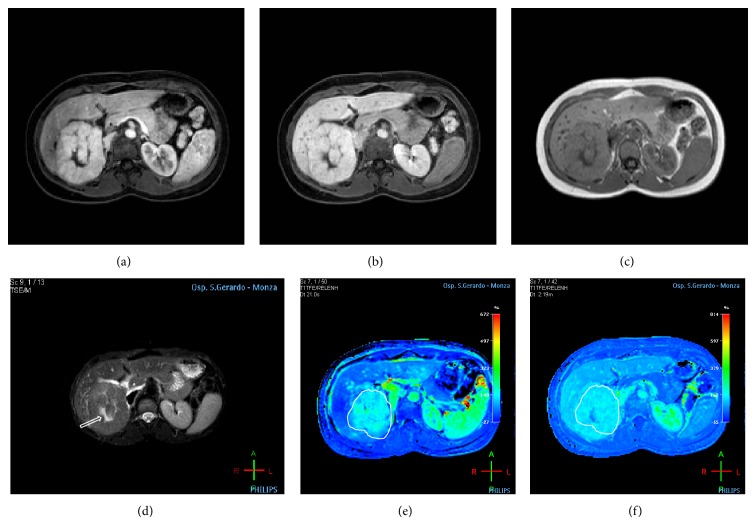
A 32-year-old woman with abdominal pain. The MR imaging shows a focal lesion in the right lobe. (a, b) Axial arterial and delayed postcontrast injection images; the lesion appears as a well-shaped hyperintense area in the arterial phase, with isointensity signal in delayed phase, with presence of hypointense central scar (arrow). (c) Axial T1-weighted in-phase echo-gradient sequence shows isointense irregular nodular area in the sixth segment. (d) Axial T2 fat-sat weighted sequence shows a rounded ill-defined nodular area, with slight intensity signal on T2-weighted images and with hyperintense scar (arrow) on the central part. (e, f) On colour perfusion maps (Relative Arterial Enhancement and Relative Late Enhancement, resp.) the lesion (ROI contouring the boundaries of the lesion) corresponds to hypervascular area.

The trend of perfusion values obtained for the different types of lesions mainly tends to depict their biological microvascular characteristics (Table 2) and to partially retrace their behavior on standard multiphasic dynamic study (Figures 1 and 2). In addition, considering the whole lesions in two main categories, (benign and malignant) the quantitative perfusion parameters showed significant differences: significantly higher values (*p* < 0.005) were obtained in benign lesions than in malignant ones (Table 3). In particular, the mean perfusion values calculated for the benign lesions are RAE 33.8%, RVE 66.03%, RLE 80.63%, ME 776.00%, MRE 86.27%, and TTP 146.95 sec. The mean perfusion values calculated for the malignant lesions are RAE 49.13%, RVE 40.54%, RLE 47.52%, ME 448.78%, MRE 48.85%, and TTP 183.79 sec.

## 4. Discussion

The liver is the most frequent cancer site after lymph glands [11]. HCC is the most common primary liver cancer and the 3rd most common cause of cancer mortality worldwide [12] but liver is also the prime target for distant metastases by digestive tract malignancies. However, also a variety of benign conditions (FNH, HA, and abscesses) can arise in the liver. Therefore, one of the main clinical needs is to accurately differentiate these lesions, trying to obtain diagnosis by employing noninvasive imaging technique and therefore avoiding biopsy.

In recent years functional imaging, including MDCT and DSCE-MRI, has been increasingly advocated in clinical practice for noninvasive assessment of vascularity of different hepatic lesions [9]. These techniques, into routine examinations, both provide excellent anatomical imaging and allow obtaining even reliable quantitative perfusion data. Considering that changes in arterial and portal venous blood flow are known to be associated with different benign and malignant focal liver lesions, the perfusion imaging might represent a potential complementary role to conventional imaging in order to improve lesions' detection and, mainly, characterization [8]. The quantitative MR perfusion imaging provides high sensitivity and high specificity information concerning the tissue microcirculation and can offer a noninvasive assessment of angiogenic activity in malignant focal liver lesions.

Perfusion MRI was firstly introduced for imaging regional and global blood flow of heart, lung, and brain [13–15]. A report on MR perfusion of the liver using gadolinium in rats was published in 1994 [16], followed by several studies including other animals and then human subjects [17]. In Materne study, tissue tracer concentration in rabbits was estimated with empiric determination of the relationship between signal intensity and T1 values with the pulse sequences used. This group subsequently used this method of perfusion MR imaging to evaluate perfusion parameters in rabbits with and without cirrhosis and also in humans [18, 19]. These studies have described and validated the use of dynamic MRI for the noninvasive quantification of hepatic perfusion. In clinical practice, the quantification of hepatic blood flow has been reported in the assessment of liver metastases and chronic liver disease and to study the systemic availability of drugs in health and disease [20–22]. In addition, assessment of hepatic perfusion parameters was employed for evaluating sinusoidal permeability changes in cirrhosis [17].

In our study we used a semiquantitative method by analyzing the signal intensity variations of the four acquired dynamic phases in order to create colorimetric maps, without adopting the arterial input. ROIs placement was basically designed in order to simplify the application of quantitative analysis in the daily clinical practice and for less time consuming alternative and to obtain quantitative data that reflect the overall component of vascularization of evaluated lesions.

To the best of our knowledge this is the first study that compared the perfusion parameters between two benign and malignant liver lesions. We found that the benign lesions presented higher values compared to the malignant ones (RAE 33.80 versus 49.13%; RVE 66.03 versus 40.54%; RLE 80.63 versus 47.52%; ME 776.00 versus 448.78%; MRE 86.27 versus 49.85%; TTP 146.95 versus 183.79%). In addition, the contrast enhancement and the perfusion values of every single type of lesion can be clustered into subtypes, also in those that present similar enhancement patterns (e.g., hypervascular lesions, such as FNH and HCC), thus offering a further complementary quantitative information that may increase the accuracy of final diagnosis.

**Figure 3:**
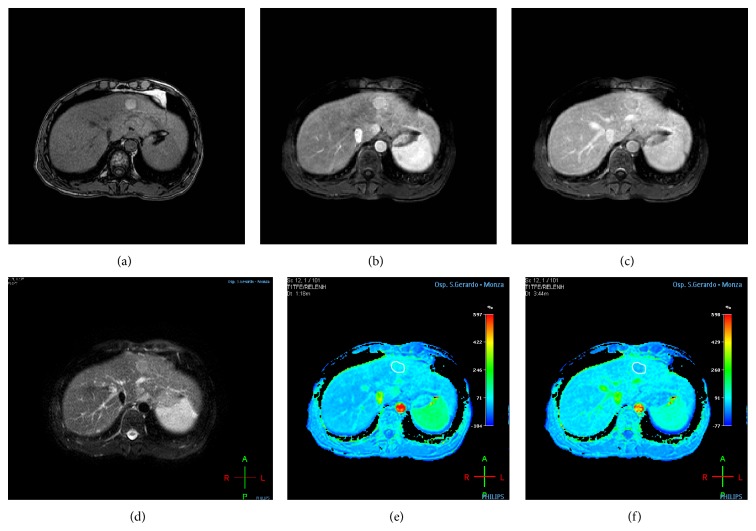
An 81-year-old man with liver cirrhosis-HCV related and with histologically proven HCC in the second segment. DCE-MR was performed to establish the presence of focal liver lesion. (a) Axial T1-weighted in-phase echo-gradient sequence shows a hyperintense nodular area in the second segment, due to presence of glycogen deposition. (b, c) Axial arterial and delayed postcontrast phase MR images show in arterial phase the presence of increased enhancement of nodular lesion, followed by washout in delayed phase. (d) Axial T2 fat-sat weighted sequence shows a weakly hyperintense nodular area in the second segment. (e, f) On colour perfusion maps (Relative Arterial Enhancement and Relative Late Enhancement, resp.) the ROIs (ROI contouring the boundaries of the lesion) were positioned on nodular lesion, and the corresponding perfusion maps show a hypervascular area, characterized by a different colour compared to the surrounding liver parenchyma with hypointensity signal in perfusion maps created for the delayed phase (f).

If we consider the single perfusion parameters, we found that the hypervascular lesions, such as FNH (Figure 2) and HCCs (Figure 3), have an elevated RAE, related to their typical hypervascularity in the arterial phases, followed by a decrease of RVE and RLE, but the washout, generally demonstrated in the venous and delayed phase of the standard dynamic study, was more represented in HCC lesions with a fast decaying of perfusion values (Table 2); angiomas show a progressive increase of all perfusion parameters in accordance with their well-known biological characteristics, with hypovascularity in the arterial phase followed by a progressive increase of vascularisation in the subsequent phases. Our study is in line with data reported by Donati et al. [3] that evaluated diffusion and perfusion MRI characteristics of FNH. All the lesions presented a quick and marked enhancement and a subsequent quick decay followed by a slowly decaying, related to the predominant arterial support of the lesions; on the other hand, the normal surrounding parenchyma presented a fast enhancement followed by a slowly decaying plateau. Therefore, the authors concluded that perfusion MRI might be an additional tool to properly diagnose FNH, providing information concerning the vascularity of the lesions.

If we instead consider metastases and HCC, we obtained a significant different cluster of quantitative perfusion pattern: homogeneously lower values for all the parameters evaluated in metastases and a higher arterial enhancement in HCC lesions. Our results confirm the quantitative analysis performed by Abdullah et al. [23], who demonstrated that perfusion MRI could be useful to characterize and differentiate HCC and colorectal liver metastases. According to this, the values of the perfusion parameters (such as RAE, ME, and MRE) in our study were significantly higher in HCC lesions than in the hypovascular metastases in accordance with the typical hypervascularity of the HCC and the hypovascularity of the metastases. In fact it is well documented that HCCs are mainly supplied by the hepatic artery while the hypovascular metastases have a prevalent portal blood flow [24].

Our study has some limitation. First of all, the number of patients, considering each liver entity, is relatively small; second the lesions characteristics were heterogeneous: the biggest lesion was about 7 cm and the smallest 1 cm, so the difference in degrees of necrosis may affect the perfusion parameters. At last, considering also cirrhotic patients, the surrounding liver that we used as reference tissue was not identical between the two groups. Furthermore, the overlap of quantitative value of single perfusion parameters may represent a bias in the functional analysis but the combination of different kinetics perfusion behaviour with the standard sequences analysis overcomes this kind of limitations.

Our feasibility study showed that employing a standard upper abdominal MRI protocol, combined with the semiquantitative analysis of dynamic contrast-enhanced T1 images, we could obtain an index of lesion's perfusion characteristics. This approach might be potentially helpful in lesions' characterization without increasing the execution time and could be also suitable for a routine use in daily clinical practice. In conclusion perfusion MRI depicting the kinetic differences in perfusion parameters among the different common types of benign and malignant liver lesions might noninvasively offer* in vivo* information about their supply and microvascular characteristics.

## Figures and Tables

**Table 1:** Descriptive parameters of acquisition protocol for the study of upper abdomen employed.

Sequences	Acquisition parameters					
	FA	Thickness	TR (msec)	TE (msec)	NSA	Matrix size
T1 in-phase AX	80°	5 mm	181	2.3	1	192 × 114
T1 out-phase AX	80°	5 mm	181	4.6	1	192 × 114
T2 TSE SPAIR AX (respiratory triggered)	90°	5 mm	432	80	2	236 × 174
T2 TSE AX	90°	5 mm	522	80	2	268 × 240
T2 TSE COR	90°	5 mm	522	80	2	268 × 240
THRIVE AX	15°	2 mm	4.1	1.97	2	176 × 160
THRIVE COR	15°	2 mm	4.1	1.97	2	188 × 188

**Note**: FA = flip angle; TR = repetition time; TE = echo time; NSA = number of signals acquired; AX = axial plane; COR = coronal plane.

**Table 2:** Summarizing table of the overall perfusion data obtained in each liver entity analyzed.

Perfusion parameters	RAE (%)	RVE (%)	RLE (%)	ME (%)	MRE (%)	TTP (sec)
Angiomas	15.98	89.17	121.12	1103.94	130.64	169.44
FNH	79.82	93.28	81.99	1100.66	98.28	89.62
Abscesses	5.6	15.1	38.8	123.4	29.9	181.8
Metastases	38.43	55.11	62.57	683.94	60.24	149.28
HCC	88.68	15.1	17.3	295.0	26.6	149.2
Cholangiocarcinoma	20.3	51.4	62.7	367.4	62.7	252.9

RAE: Relative Arterial Enhancement; RVE: Relative Venous Enhancement; RLE: Relative Late Enhancement; ME: Maximum Enhancement; MRE: Maximum Relative Enhancement; TTP: time to peak.

**Table 3:** Mean values of perfusion parameters obtained for benign and malignant lesions.

Perfusion values	RAE (%)	RVE (%)	RLE (%)	ME (%)	MRE (%)	TTP (sec)
Benign lesions	33.8	66.03	80.63	776.00	86.27	146.95
Malignant lesions	49.13	40.54	47.52	448.78	49.85	183.79
*p* value	0.30	0.01	0.10	0.05	0.01	0.17

*p*  value <0.05 calculated with Mann-Whitney test indicated a statistically significant difference.
